# *N*-(4-Methyl­phen­yl)-3-nitro­pyridin-2-amine^1^

**DOI:** 10.1107/S1600536810033040

**Published:** 2010-08-25

**Authors:** Mardia Aina Aznan Akhmad, Zanariah Abdullah, Zainal A. Fairuz, Seik Weng Ng, Edward R. T. Tiekink

**Affiliations:** a Department of Chemistry, University of Malaya, 50603 Kuala Lumpur, Malaysia

## Abstract

Two independent mol­ecules comprise the asymmetric unit of the title compound, C_12_H_11_N_3_O_2_. These differ in terms of the relative orientations of the benzene rings as seen in the respective dihedral angles formed between the pyridine and benzene rings [17.42 (16) and 34.64 (16)°]. Both mol­ecules are twisted about the amine–tolyl N—C bonds [respective torsion angles = 22.3 (5) and 35.9 (5)°] but only about the amine–pyridine N—C bond in the first independent mol­ecule [respective torsion angles = −11.7 (5) and 0.8 (5)°]. Intra­molecular N—H⋯O hydrogen bonds preclude the amine H atoms from forming significant inter­molecular inter­actions. The crystal packing features inter­molecular C—H⋯O and C—H⋯π and π–π [centroid–centroid distance: pyridine–benzene = 3.6442 (19) Å and pyridine–pyridine = 3.722 (2) Å] contacts.

## Related literature

For background to the fluorescence properties of compounds related to the title compound, see: Kawai *et al.* (2001 ▶); Abdullah (2005 ▶). For the structures of related pyrimidine amine derivatives, see: Badaruddin *et al.*, (2009 ▶); Fairuz *et al.* (2010 ▶).
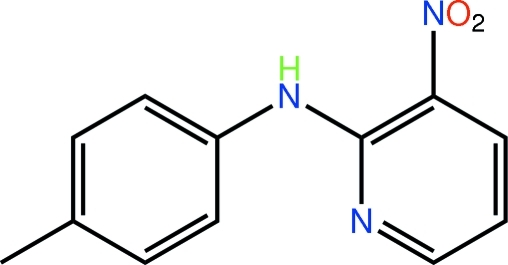


## Experimental

### 

#### Crystal data

C_12_H_11_N_3_O_2_
*M* *_r_* = 229.24
Monoclinic, 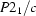

*a* = 10.6557 (12) Å
*b* = 7.1415 (8) Å
*c* = 27.958 (3) Å
β = 91.310 (2)°
*V* = 2127.0 (4) Å^3^
*Z* = 8
Mo *K*α radiation
μ = 0.10 mm^−1^
*T* = 100 K
0.35 × 0.35 × 0.05 mm

#### Data collection

Bruker SMART APEX diffractometer
13178 measured reflections
4804 independent reflections
3344 reflections with *I* > 2σ(*I*)
*R*_int_ = 0.045

#### Refinement

*R*[*F* ^2^ > 2σ(*F* ^2^)] = 0.048
*wR*(*F* ^2^) = 0.146
*S* = 1.04
4804 reflections
317 parameters
2 restraints
H atoms treated by a mixture of independent and constrained refinement
Δρ_max_ = 0.30 e Å^−3^
Δρ_min_ = −0.28 e Å^−3^

### 

Data collection: *APEX2* (Bruker, 2009 ▶); cell refinement: *SAINT* (Bruker, 2009 ▶); data reduction: *SAINT*; program(s) used to solve structure: *SHELXS97* (Sheldrick, 2008 ▶); program(s) used to refine structure: *SHELXL97* (Sheldrick, 2008 ▶); molecular graphics: *ORTEP-3* (Farrugia, 1997 ▶), *DIAMOND* (Brandenburg, 2006 ▶) and Qmol (Gans & Shalloway, 2001 ▶); software used to prepare material for publication: *publCIF* (Westrip, 2010 ▶).

## Supplementary Material

Crystal structure: contains datablocks global, I. DOI: 10.1107/S1600536810033040/hg2703sup1.cif

Structure factors: contains datablocks I. DOI: 10.1107/S1600536810033040/hg2703Isup2.hkl

Additional supplementary materials:  crystallographic information; 3D view; checkCIF report

## Figures and Tables

**Table 1:** Hydrogen-bond geometry (Å, °) *Cg*1 is the centroid of the C6–C11 ring.

*D*—H⋯*A*	*D*—H	H⋯*A*	*D*⋯*A*	*D*—H⋯*A*
N3—H3⋯O1	0.87 (3)	1.94 (3)	2.630 (3)	136 (3)
N6—H6⋯O3	0.88 (4)	1.93 (4)	2.639 (3)	137 (3)
N3—H3⋯N1	0.87 (3)	2.55 (3)	2.932 (4)	108 (2)
N6—H6⋯N4	0.88 (4)	2.54 (4)	2.942 (4)	109 (2)
C14—H14⋯O2^i^	0.95	2.39	3.199 (4)	143
C4—H4⋯*Cg*1^ii^	0.95	2.90	3.604 (4)	132
C12—H12b⋯*Cg*1^iii^	0.98	2.80	3.654 (4)	146

Symmetry codes: (i) 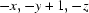; (ii) 
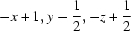
; (iii) 
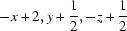
.

**Fig. 1.**
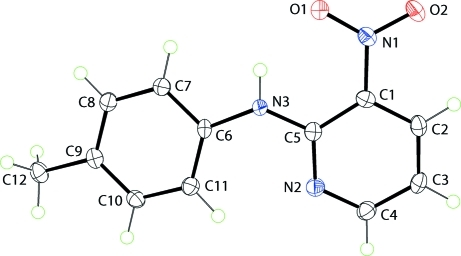
The molecular structure of the first independent molecule in (I) showing the atom-labelling scheme and displacement ellipsoids at the 50% probability level.

**Fig. 2.**
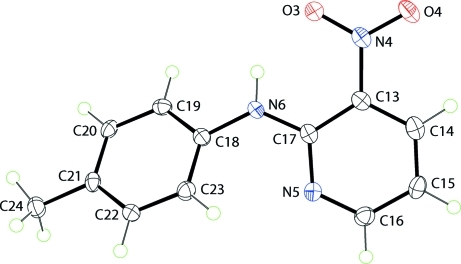
The molecular structure of the second independent molecule in (I) showing the atom-labelling scheme and displacement ellipsoids at the 50% probability level.

**Fig. 3.**
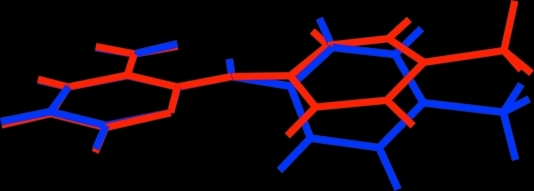
Overlay diagram of the first independent molecule (shown in red) and the second independent molecule (shown in blue).

**Fig. 4.**
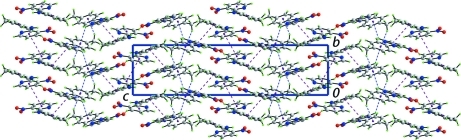
Unit-cell contents for (I) shown in projection down the a axis. The C–H···π and π–π contacts are shown as blue and purple dashed lines, respectively. The C–H···O interactions are largely obscured in this projection.

## supplementary crystallographic information

### Comment

Studies of pyridine and pyrimidine derivatives related to the title compound are of interest owing to their putative fluorescence properties (Kawai *et al.* 2001; Abdullah, 2005). As a continuation of structural studies on this class of *N*-heterocycles (Badaruddin *et al.*, 2009; Fairuz *et al.*, 2010), the title compound, (I), was investigated.

Two independent molecules comprise the asymmetric unit of (I), Figs 1 and 2. While the geometric parameters are in close agreement [r.m.s. deviation of bond distances and angles = 0.0080 Å and 0.849 °, respectively], these differ non-trivially in their conformations. So, while the pyridine groups are virtually super-imposable, the benzene rings are not, Fig. 3. This difference is quantified in the dihedral angles formed between the pyridine and benzene rings, *i.e*. N2,C1–C5/C6–C11 = 17.42 (16) ° and N5,C13–C17/C18–C23 = 34.64 (16) °. These indicate that there are also twists in the molecules as evidenced by the C6–N3–C5–N2 torsion angle of -11.7 (5) ° and, especially, the C5–N3–C6–C11 torsion angle of 22.3 (5) °. The equivalent torsion angles for the second independent molecule of C18–N6–C17–N5 = 0.8 (5) ° and C17–N6–C18–C23 = 35.9 (5) ° also highlight the differences between the molecules. The nitro groups are co-planar with the pyridine rings to which they are connected as indicated by the O1–N1–C1–C5 and O3–N4–C13–C17 torsion angles of 1.5 (5) and -1.1 (5) °, respectively. Close intramolecular N–H···O and C–H···N interactions are noted, Table 1.

The most notable intermolecular interactions in the crystal structure are of the type C–H···O [which connect the molecules comprising the asymmetric unit], C–H···π and π–π [ring centroid(N2,C1–C5)···centroid(C18–C23) = 3.6442 (19) Å and ring centroid(N5,C13–C17)···centroid(N5,C13–C17)^i^ = 3.722 (2) Å for *i*: -*x*, 1 - *y*, -*z*] contacts. These serve to connect molecules into the three-dimensional structure, Fig. 4.

### Experimental

2-Chloro-3-nitro-pyridine (0.7899 g, 0.005 mol) and *p*-toluidine (0.536 g, 0.005 mol) were refluxed in 5 ml ethanol for 5.5 h at 351 K. The mixture was cooled. The residue was then dissolved in a minimum volume of water (10 ml) and extracted with ether (3 *x* 10 ml). The ethereal layer was washed with water and dried over anhydrous sodium sulfate. Evaporation gave a reddish solid and recrystallization using ethyl acetate yielded red crystals.

### Refinement

Carbon-bound H-atoms were placed in calculated positions (C—H 0.95 to 0.98 Å) and were included in the refinement in the riding model approximation, with *U*_iso_(H) set to 1.2 to 1.5*U*_equiv_(C). The N-bound H-atoms were located in a difference Fourier map but were were refined with a distance restraint of N–H = 0.86±0.01 Å, and with unrestricted *U*_iso_(H).

### Crystal data

**Table:**

C_12_H_11_N_3_O_2_	*F*(000) = 960
*M**_r_* = 229.24	*D*_x_ = 1.432 Mg m^−^^3^
Monoclinic, *P*2_1_/*c*	Mo *K*α radiation, λ = 0.71073 Å
Hall symbol: -P 2ybc	Cell parameters from 2221 reflections
*a* = 10.6557 (12) Å	θ = 3.0–27.8°
*b* = 7.1415 (8) Å	µ = 0.10 mm^−^^1^
*c* = 27.958 (3) Å	*T* = 100 K
β = 91.310 (2)°	Plate, red
*V* = 2127.0 (4) Å^3^	0.35 × 0.35 × 0.05 mm
*Z* = 8	

### Data collection

**Table:**

Bruker SMART APEX diffractometer	3344 reflections with *I* > 2σ(*I*)
Radiation source: fine-focus sealed tube	*R*_int_ = 0.045
graphite	θ_max_ = 27.5°, θ_min_ = 1.5°
ω scans	*h* = −13→11
13178 measured reflections	*k* = −8→9
4804 independent reflections	*l* = −36→36

### Refinement

**Table:**

Refinement on *F*^2^	Primary atom site location: structure-invariant direct methods
Least-squares matrix: full	Secondary atom site location: difference Fourier map
*R*[*F*^2^ > 2σ(*F*^2^)] = 0.048	Hydrogen site location: inferred from neighbouring sites
*wR*(*F*^2^) = 0.146	H atoms treated by a mixture of independent and constrained refinement
*S* = 1.04	*w* = 1/[σ^2^(*F*_o_^2^) + (0.0685*P*)^2^ + 0.8145*P*] where *P* = (*F*_o_^2^ + 2*F*_c_^2^)/3
4804 reflections	(Δ/σ)_max_ = 0.001
317 parameters	Δρ_max_ = 0.30 e Å^−^^3^
2 restraints	Δρ_min_ = −0.28 e Å^−^^3^

### Special details

**Table:**

Geometry. All e.s.d.'s (except the e.s.d. in the dihedral angle between two l.s. planes) are estimated using the full covariance matrix. The cell e.s.d.'s are taken into account individually in the estimation of e.s.d.'s in distances, angles and torsion angles; correlations between e.s.d.'s in cell parameters are only used when they are defined by crystal symmetry. An approximate (isotropic) treatment of cell e.s.d.'s is used for estimating e.s.d.'s involving l.s. planes.
Refinement. Refinement of *F*^2^ against ALL reflections. The weighted *R*-factor *wR* and goodness of fit *S* are based on *F*^2^, conventional *R*-factors *R* are based on *F*, with *F* set to zero for negative *F*^2^. The threshold expression of *F*^2^ > σ(*F*^2^) is used only for calculating *R*-factors(gt) *etc*. and is not relevant to the choice of reflections for refinement. *R*-factors based on *F*^2^ are statistically about twice as large as those based on *F*, and *R*- factors based on ALL data will be even larger.

### Fractional atomic coordinates and isotropic or equivalent isotropic displacement parameters (Å^2^)

**Table:**

	*x*	*y*	*z*	*U*_iso_*/*U*_eq_	
O1	0.5737 (2)	0.6059 (3)	0.07334 (8)	0.0224 (6)	
O2	0.3802 (2)	0.6139 (4)	0.04820 (8)	0.0265 (6)	
O3	0.1554 (2)	0.1650 (4)	−0.07299 (8)	0.0286 (6)	
O4	−0.0348 (2)	0.2379 (4)	−0.09457 (8)	0.0286 (6)	
N1	0.4595 (3)	0.5848 (4)	0.08003 (9)	0.0186 (6)	
N2	0.4564 (3)	0.4326 (4)	0.20794 (9)	0.0193 (6)	
N3	0.6278 (2)	0.5089 (4)	0.16220 (9)	0.0163 (6)	
N4	0.0469 (3)	0.2118 (4)	−0.06319 (9)	0.0207 (6)	
N5	0.0665 (3)	0.2476 (4)	0.06993 (9)	0.0200 (6)	
N6	0.2219 (3)	0.1555 (4)	0.01848 (9)	0.0191 (6)	
C1	0.4176 (3)	0.5224 (4)	0.12647 (10)	0.0171 (7)	
C2	0.2897 (3)	0.4973 (5)	0.13170 (11)	0.0195 (7)	
H2	0.2331	0.5196	0.1056	0.023*	
C3	0.2457 (3)	0.4396 (5)	0.17510 (11)	0.0206 (7)	
H3A	0.1586	0.4213	0.1798	0.025*	
C4	0.3327 (3)	0.4092 (5)	0.21164 (11)	0.0211 (7)	
H4	0.3022	0.3687	0.2416	0.025*	
C5	0.5016 (3)	0.4879 (4)	0.16576 (11)	0.0161 (7)	
C6	0.7235 (3)	0.5118 (4)	0.19792 (11)	0.0154 (7)	
C7	0.8350 (3)	0.5993 (4)	0.18500 (11)	0.0169 (7)	
H7	0.8427	0.6476	0.1535	0.020*	
C8	0.9339 (3)	0.6166 (5)	0.21729 (11)	0.0185 (7)	
H8	1.0089	0.6759	0.2076	0.022*	
C9	0.9263 (3)	0.5489 (5)	0.26396 (11)	0.0179 (7)	
C10	0.8168 (3)	0.4554 (5)	0.27559 (11)	0.0184 (7)	
H10	0.8106	0.4034	0.3067	0.022*	
C11	0.7155 (3)	0.4345 (5)	0.24348 (11)	0.0184 (7)	
H11	0.6424	0.3686	0.2526	0.022*	
C12	1.0323 (3)	0.5790 (5)	0.29973 (11)	0.0224 (7)	
H12A	1.0097	0.5253	0.3306	0.034*	
H12B	1.0478	0.7135	0.3035	0.034*	
H12C	1.1083	0.5178	0.2883	0.034*	
C13	0.0149 (3)	0.2401 (5)	−0.01373 (11)	0.0183 (7)	
C14	−0.1058 (3)	0.3001 (5)	−0.00485 (12)	0.0212 (7)	
H14	−0.1649	0.3152	−0.0305	0.025*	
C15	−0.1398 (3)	0.3376 (5)	0.04115 (12)	0.0220 (7)	
H15	−0.2214	0.3817	0.0482	0.026*	
C16	−0.0491 (3)	0.3079 (5)	0.07704 (12)	0.0222 (7)	
H16	−0.0720	0.3328	0.1091	0.027*	
C17	0.1032 (3)	0.2129 (4)	0.02481 (11)	0.0172 (7)	
C18	0.3186 (3)	0.1221 (5)	0.05334 (11)	0.0173 (7)	
C19	0.4414 (3)	0.1571 (5)	0.03961 (11)	0.0189 (7)	
H19	0.4568	0.2063	0.0087	0.023*	
C20	0.5413 (3)	0.1203 (5)	0.07102 (11)	0.0188 (7)	
H20	0.6246	0.1423	0.0610	0.023*	
C21	0.5218 (3)	0.0519 (5)	0.11668 (11)	0.0179 (7)	
C22	0.3992 (3)	0.0150 (5)	0.12938 (11)	0.0188 (7)	
H22	0.3840	−0.0340	0.1603	0.023*	
C23	0.2979 (3)	0.0474 (5)	0.09839 (11)	0.0201 (7)	
H23	0.2151	0.0187	0.1079	0.024*	
C24	0.6290 (3)	0.0200 (5)	0.15168 (12)	0.0242 (8)	
H24A	0.7084	0.0220	0.1347	0.036*	
H24B	0.6189	−0.1019	0.1672	0.036*	
H24C	0.6296	0.1190	0.1759	0.036*	
H3	0.652 (3)	0.552 (5)	0.1350 (8)	0.022 (10)*	
H6	0.242 (3)	0.153 (6)	−0.0118 (14)	0.031 (11)*	

### Atomic displacement parameters (Å^2^)

**Table:**

	*U*^11^	*U*^22^	*U*^33^	*U*^12^	*U*^13^	*U*^23^
O1	0.0175 (13)	0.0287 (14)	0.0210 (11)	−0.0020 (10)	−0.0014 (9)	0.0037 (10)
O2	0.0236 (13)	0.0349 (15)	0.0206 (11)	0.0030 (11)	−0.0063 (10)	0.0061 (10)
O3	0.0235 (14)	0.0429 (16)	0.0195 (12)	0.0097 (12)	−0.0008 (10)	−0.0016 (11)
O4	0.0288 (14)	0.0353 (15)	0.0212 (12)	−0.0007 (12)	−0.0093 (10)	0.0011 (10)
N1	0.0209 (15)	0.0172 (15)	0.0178 (13)	0.0010 (12)	−0.0025 (11)	0.0008 (10)
N2	0.0189 (15)	0.0212 (15)	0.0179 (13)	−0.0001 (12)	0.0012 (11)	−0.0003 (11)
N3	0.0131 (14)	0.0217 (15)	0.0140 (12)	−0.0016 (11)	−0.0009 (10)	0.0025 (10)
N4	0.0220 (16)	0.0199 (15)	0.0200 (13)	−0.0018 (12)	−0.0040 (11)	0.0017 (11)
N5	0.0177 (15)	0.0224 (15)	0.0198 (13)	0.0022 (12)	0.0000 (11)	0.0008 (11)
N6	0.0172 (15)	0.0254 (16)	0.0147 (13)	0.0025 (12)	−0.0024 (11)	0.0008 (11)
C1	0.0215 (18)	0.0143 (16)	0.0156 (14)	0.0000 (13)	0.0000 (12)	−0.0003 (12)
C2	0.0172 (17)	0.0174 (17)	0.0236 (16)	0.0017 (13)	−0.0046 (13)	−0.0006 (13)
C3	0.0127 (17)	0.0218 (18)	0.0274 (17)	−0.0001 (14)	0.0007 (13)	−0.0011 (14)
C4	0.0199 (18)	0.0232 (18)	0.0203 (15)	−0.0021 (14)	0.0024 (13)	−0.0003 (13)
C5	0.0170 (17)	0.0141 (16)	0.0173 (14)	−0.0002 (13)	−0.0004 (12)	−0.0032 (11)
C6	0.0116 (16)	0.0171 (16)	0.0173 (14)	0.0012 (13)	−0.0018 (12)	−0.0027 (12)
C7	0.0172 (17)	0.0164 (16)	0.0171 (14)	0.0009 (13)	0.0009 (12)	−0.0004 (12)
C8	0.0167 (17)	0.0159 (17)	0.0229 (16)	0.0000 (13)	0.0002 (13)	0.0001 (12)
C9	0.0185 (17)	0.0151 (16)	0.0198 (15)	0.0028 (13)	−0.0025 (12)	−0.0041 (12)
C10	0.0198 (18)	0.0206 (17)	0.0149 (14)	0.0042 (14)	0.0019 (12)	0.0002 (12)
C11	0.0171 (17)	0.0192 (17)	0.0189 (15)	0.0007 (14)	0.0014 (12)	0.0001 (13)
C12	0.0232 (19)	0.0211 (18)	0.0227 (16)	−0.0005 (15)	−0.0064 (14)	−0.0014 (13)
C13	0.0208 (18)	0.0162 (16)	0.0180 (15)	−0.0029 (14)	−0.0017 (12)	0.0009 (12)
C14	0.0195 (18)	0.0172 (17)	0.0265 (17)	−0.0050 (14)	−0.0046 (13)	0.0044 (13)
C15	0.0148 (17)	0.0204 (18)	0.0309 (18)	−0.0001 (14)	0.0025 (14)	0.0040 (14)
C16	0.0219 (18)	0.0226 (18)	0.0222 (16)	0.0002 (15)	0.0038 (13)	0.0028 (13)
C17	0.0181 (17)	0.0144 (16)	0.0190 (15)	−0.0022 (13)	−0.0026 (12)	0.0020 (12)
C18	0.0205 (18)	0.0145 (16)	0.0168 (14)	0.0019 (13)	−0.0017 (12)	−0.0019 (12)
C19	0.0236 (18)	0.0169 (17)	0.0162 (14)	0.0005 (14)	0.0032 (12)	0.0010 (12)
C20	0.0124 (16)	0.0218 (18)	0.0223 (16)	0.0000 (13)	0.0024 (12)	−0.0021 (13)
C21	0.0172 (17)	0.0150 (16)	0.0214 (15)	0.0029 (13)	−0.0036 (13)	−0.0040 (12)
C22	0.0186 (17)	0.0214 (18)	0.0165 (14)	0.0020 (14)	0.0011 (12)	0.0030 (12)
C23	0.0167 (17)	0.0219 (18)	0.0217 (15)	−0.0006 (14)	0.0023 (13)	0.0030 (13)
C24	0.0235 (19)	0.0256 (19)	0.0232 (16)	0.0044 (15)	−0.0049 (14)	−0.0019 (14)

### Geometric parameters (Å, °)

**Table:**

O1—N1	1.245 (3)	C9—C10	1.389 (5)
O2—N1	1.230 (3)	C9—C12	1.507 (4)
O3—N4	1.240 (4)	C10—C11	1.396 (4)
O4—N4	1.236 (3)	C10—H10	0.9500
N1—C1	1.452 (4)	C11—H11	0.9500
N2—C4	1.335 (4)	C12—H12A	0.9800
N2—C5	1.343 (4)	C12—H12B	0.9800
N3—C5	1.359 (4)	C12—H12C	0.9800
N3—C6	1.412 (4)	C13—C14	1.383 (5)
N3—H3	0.867 (15)	C13—C17	1.428 (4)
N4—C13	1.446 (4)	C14—C15	1.370 (5)
N5—C16	1.324 (4)	C14—H14	0.9500
N5—C17	1.352 (4)	C15—C16	1.393 (5)
N6—C17	1.345 (4)	C15—H15	0.9500
N6—C18	1.422 (4)	C16—H16	0.9500
N6—H6	0.88 (4)	C18—C23	1.390 (4)
C1—C2	1.386 (5)	C18—C19	1.394 (4)
C1—C5	1.422 (4)	C19—C20	1.390 (4)
C2—C3	1.374 (4)	C19—H19	0.9500
C2—H2	0.9500	C20—C21	1.387 (4)
C3—C4	1.380 (4)	C20—H20	0.9500
C3—H3A	0.9500	C21—C22	1.388 (4)
C4—H4	0.9500	C21—C24	1.505 (4)
C6—C11	1.393 (4)	C22—C23	1.388 (4)
C6—C7	1.397 (4)	C22—H22	0.9500
C7—C8	1.377 (4)	C23—H23	0.9500
C7—H7	0.9500	C24—H24A	0.9800
C8—C9	1.396 (4)	C24—H24B	0.9800
C8—H8	0.9500	C24—H24C	0.9800
			
O2—N1—O1	121.9 (3)	C10—C11—H11	120.5
O2—N1—C1	118.6 (3)	C9—C12—H12A	109.5
O1—N1—C1	119.5 (2)	C9—C12—H12B	109.5
C4—N2—C5	118.6 (3)	H12A—C12—H12B	109.5
C5—N3—C6	130.6 (3)	C9—C12—H12C	109.5
C5—N3—H3	115 (2)	H12A—C12—H12C	109.5
C6—N3—H3	113 (2)	H12B—C12—H12C	109.5
O4—N4—O3	121.9 (3)	C14—C13—C17	120.4 (3)
O4—N4—C13	118.7 (3)	C14—C13—N4	117.1 (3)
O3—N4—C13	119.4 (3)	C17—C13—N4	122.5 (3)
C16—N5—C17	119.3 (3)	C15—C14—C13	119.7 (3)
C17—N6—C18	129.0 (3)	C15—C14—H14	120.2
C17—N6—H6	113 (3)	C13—C14—H14	120.2
C18—N6—H6	118 (3)	C14—C15—C16	116.9 (3)
C2—C1—C5	119.9 (3)	C14—C15—H15	121.5
C2—C1—N1	117.2 (3)	C16—C15—H15	121.5
C5—C1—N1	122.9 (3)	N5—C16—C15	125.0 (3)
C3—C2—C1	119.2 (3)	N5—C16—H16	117.5
C3—C2—H2	120.4	C15—C16—H16	117.5
C1—C2—H2	120.4	N6—C17—N5	118.1 (3)
C2—C3—C4	117.7 (3)	N6—C17—C13	123.2 (3)
C2—C3—H3A	121.2	N5—C17—C13	118.6 (3)
C4—C3—H3A	121.2	C23—C18—C19	119.2 (3)
N2—C4—C3	124.8 (3)	C23—C18—N6	123.9 (3)
N2—C4—H4	117.6	C19—C18—N6	116.8 (3)
C3—C4—H4	117.6	C20—C19—C18	120.2 (3)
N2—C5—N3	118.2 (3)	C20—C19—H19	119.9
N2—C5—C1	119.8 (3)	C18—C19—H19	119.9
N3—C5—C1	122.0 (3)	C21—C20—C19	121.3 (3)
C11—C6—C7	119.0 (3)	C21—C20—H20	119.3
C11—C6—N3	125.7 (3)	C19—C20—H20	119.3
C7—C6—N3	115.4 (3)	C22—C21—C20	117.7 (3)
C8—C7—C6	120.8 (3)	C22—C21—C24	120.7 (3)
C8—C7—H7	119.6	C20—C21—C24	121.6 (3)
C6—C7—H7	119.6	C21—C22—C23	122.1 (3)
C7—C8—C9	121.5 (3)	C21—C22—H22	118.9
C7—C8—H8	119.3	C23—C22—H22	118.9
C9—C8—H8	119.3	C22—C23—C18	119.5 (3)
C10—C9—C8	116.9 (3)	C22—C23—H23	120.2
C10—C9—C12	122.3 (3)	C18—C23—H23	120.2
C8—C9—C12	120.8 (3)	C21—C24—H24A	109.5
C9—C10—C11	122.8 (3)	C21—C24—H24B	109.5
C9—C10—H10	118.6	H24A—C24—H24B	109.5
C11—C10—H10	118.6	C21—C24—H24C	109.5
C6—C11—C10	118.9 (3)	H24A—C24—H24C	109.5
C6—C11—H11	120.5	H24B—C24—H24C	109.5
			
O2—N1—C1—C2	0.8 (4)	O4—N4—C13—C14	−1.7 (4)
O1—N1—C1—C2	−178.6 (3)	O3—N4—C13—C14	177.3 (3)
O2—N1—C1—C5	−179.0 (3)	O4—N4—C13—C17	179.9 (3)
O1—N1—C1—C5	1.5 (5)	O3—N4—C13—C17	−1.1 (5)
C5—C1—C2—C3	0.3 (5)	C17—C13—C14—C15	1.4 (5)
N1—C1—C2—C3	−179.6 (3)	N4—C13—C14—C15	−177.0 (3)
C1—C2—C3—C4	−0.1 (5)	C13—C14—C15—C16	−1.5 (5)
C5—N2—C4—C3	−0.7 (5)	C17—N5—C16—C15	0.9 (5)
C2—C3—C4—N2	0.3 (5)	C14—C15—C16—N5	0.4 (5)
C4—N2—C5—N3	−179.1 (3)	C18—N6—C17—N5	0.8 (5)
C4—N2—C5—C1	0.8 (5)	C18—N6—C17—C13	−179.7 (3)
C6—N3—C5—N2	−11.7 (5)	C16—N5—C17—N6	178.6 (3)
C6—N3—C5—C1	168.4 (3)	C16—N5—C17—C13	−0.9 (5)
C2—C1—C5—N2	−0.6 (5)	C14—C13—C17—N6	−179.7 (3)
N1—C1—C5—N2	179.2 (3)	N4—C13—C17—N6	−1.4 (5)
C2—C1—C5—N3	179.3 (3)	C14—C13—C17—N5	−0.2 (5)
N1—C1—C5—N3	−0.8 (5)	N4—C13—C17—N5	178.1 (3)
C5—N3—C6—C11	22.3 (5)	C17—N6—C18—C23	35.9 (5)
C5—N3—C6—C7	−158.4 (3)	C17—N6—C18—C19	−148.1 (3)
C11—C6—C7—C8	−2.8 (5)	C23—C18—C19—C20	−1.0 (5)
N3—C6—C7—C8	177.8 (3)	N6—C18—C19—C20	−177.3 (3)
C6—C7—C8—C9	−0.5 (5)	C18—C19—C20—C21	−1.3 (5)
C7—C8—C9—C10	3.1 (5)	C19—C20—C21—C22	2.3 (5)
C7—C8—C9—C12	−176.3 (3)	C19—C20—C21—C24	−177.1 (3)
C8—C9—C10—C11	−2.6 (5)	C20—C21—C22—C23	−1.1 (5)
C12—C9—C10—C11	176.8 (3)	C24—C21—C22—C23	178.3 (3)
C7—C6—C11—C10	3.2 (5)	C21—C22—C23—C18	−1.1 (5)
N3—C6—C11—C10	−177.4 (3)	C19—C18—C23—C22	2.2 (5)
C9—C10—C11—C6	−0.6 (5)	N6—C18—C23—C22	178.1 (3)

### Hydrogen-bond geometry (Å, °)

**Table:**

Cg1 is the centroid of the C6–C11 ring.

**Table:**

*D*—H···*A*	*D*—H	H···*A*	*D*···*A*	*D*—H···*A*
N3—H3···O1	0.87 (3)	1.94 (3)	2.630 (3)	136 (3)
N6—H6···O3	0.88 (4)	1.93 (4)	2.639 (3)	137 (3)
N3—H3···N1	0.87 (3)	2.55 (3)	2.932 (4)	108 (2)
N6—H6···N4	0.88 (4)	2.54 (4)	2.942 (4)	109 (2)
C14—H14···O2^i^	0.95	2.39	3.199 (4)	143
C4—H4···Cg1^ii^	0.95	2.90	3.604 (4)	132
C12—H12b···Cg1^iii^	0.98	2.80	3.654 (4)	146

Symmetry codes: (i) −*x*, −*y*+1, −*z*; (ii) −*x*+1, *y*−1/2, −*z*+1/2; (iii) −*x*+2, *y*+1/2, −*z*+1/2.
